# Role of the Default Mode Network in Cognitive Transitions

**DOI:** 10.1093/cercor/bhy167

**Published:** 2018-07-27

**Authors:** Verity Smith, Daniel J Mitchell, John Duncan

**Affiliations:** 1 Medical Research Council Cognition and Brain Sciences Unit, University of Cambridge, Cambridge, UK; 2 Department of Experimental Psychology, University of Oxford, Oxford, UK

**Keywords:** cognitive control, fMRI, task switching

## Abstract

A frequently repeated finding is that the default mode network (DMN) shows activation decreases during externally focused tasks. This finding has led to an emphasis in DMN research on internally focused self-relevant thought processes. A recent study, in contrast, implicates the DMN in substantial externally focused task switches. Using functional magnetic resonance imaging, we scanned 24 participants performing a task switch experiment. Whilst replicating previous DMN task switch effects, we also found large DMN increases for brief rests as well as task restarts after rest. Our findings are difficult to explain using theories strictly linked to internal or self-directed cognition. In line with principal results from the literature, we suggest that the DMN encodes scene, episode or context, by integrating spatial, self-referential, and temporal information. Context representations are strong at rest, but rereference to context also occurs at major cognitive transitions.

## Introduction

The default mode network (DMN) is one of the most robust discoveries of neuroimaging. Default mode regions—prominently including parts of medial frontal cortex, posterior cingulate, posterolateral parietal cortex, retrosplenial cortex, and hippocampal formation—show strong functional connectivity at rest (Fransson and Marrelec 2008; Greicius et al. 2003; Andrews-Hanna et al. 2010) and commonly increase or decrease activity together across a wide range of cognitive manipulations (Shulman et al. 1997; Spreng et al. 2009). Despite these robust findings, the functional significance of the DMN remains unclear.

Following the early work of Shulman et al. (1997), one much-replicated result is decreased DMN activity in many tasks compared with rest. These decreases complement common patterns of task-related increase in other networks, including “dorsal attention” (Corbetta and Shulman 2002) and “multiple-demand” or MD networks (Duncan and Owen 2000; Duncan 2010). Stronger activity during rest compared with focused task performance led early on to the proposal that the DMN is involved in internally generated cognition, including mind-wandering (Mason et al. 2007; Christoff et al. 2009) and self-related thought (Johnson et al. 2002; D’Argembeau et al. 2005; Andrews-Hanna et al. 2010). Subsequent findings lend support to this emphasis on internally directed and self-relevant cognition, including DMN activation during autobiographical memory recollection (Diana et al. 2007; Schacter et al. 2007; Vilberg and Rugg 2012), imagining possible future events (Addis et al. 2007; Andrews-Hanna et al. 2010; Spreng et al. 2010), making self-referential judgments (Johnson et al. 2002; D’Argembeau et al. 2005), imagining routes (Kumaran and Maguire 2005; Spiers and Maguire 2007; Howard et al. 2014; Balaguer et al. 2016), etc. A broad suggestion is that the DMN creates internal scenes (Hassabis and Maguire 2007, 2009), episodes (Addis et al. 2007; Buckner and Carroll 2007), or contexts (Bar 2007, 2009; Ranganath and Ritchey 2012), allowing cognition to escape from the constraints of the present environment (Smallwood et al. 2013; Konishi et al. 2015). Such an internal scene or context might include spatial, temporal, social and perhaps other elements. Both imaging and animal experiments, for example, link parts of the DMN (especially posterior cingulate, retrosplenial cortex, hippocampus) to spatial representation and navigation (Vann and Aggleton 2002; Burwell et al. 2004; Spiers and Maguire 2007; Bachevalier and Nemanic 2008; Doeller et al. 2010; Jacobs et al. 2013; Howard et al. 2014; see Bird and Burgess 2008 for a review). Medial frontal and posterior parietal regions show strong activity linked to social cognition, including consideration of others’ mental states (Frith and Frith 2003; Kumaran and Maguire 2005; Amodio and Frith 2006). Much DMN activity is also linked to time, as in recollection and future planning (Addis et al. 2007; Andrews-Hanna et al. 2010).

Despite this broad emphasis on the role of the DMN in internally directed cognition, it seems likely that much would be shared between processing of current and internally constructed cognitive contexts. In line with this, Ranganath and Ritchey (2012) suggest that a posterior medial system, including many DMN regions, is centrally important for the representation and application of situational models which could reflect either current or imagined contexts. Perhaps consistent with such a view, some findings show a role for the DMN in externally as well as internally focused cognition (Lee et al. 2005; Christoff et al. 2009; Baldassano et al. 2016; Chen et al. 2017; see discussion in Margulies and Smallwood 2017). Of most relevance to the current work, a study by Crittenden et al. (2015) implicated the DMN in externally focused task switching. In this study, participants were asked to perform a yes/no task following a task rule cued for by the color of frame surrounding the imperative stimuli. Two tasks were associated with each of 3 stimulus domains (pictures, words and shapes). Tasks were presented sequentially in a pseudorandom order to create task stay trials (where the current task is the same as the previous task), within-domain switch trials (where the current task involves the same stimulus domain as the previous task) and between-domain switch trials (where the current task involves a different domain of stimulus compared with the previous task). Following Andrews-Hanna et al. (2010), Crittenden et al. (2015) divided the DMN into 3 subnetworks, “core” (anteromedial frontal cortex and posterior cingulate), “medial temporal lobe (MTL)” (retrosplenial, parahappicompal, and hippocampal cortex, along with posterior inferior parietal and ventromedial frontal cortex) and “dorsomedial prefrontal (dmPFC)” (dorsomedial prefrontal cortex accompanied by regions in lateral temporal lobe and temporoparietal junction). Contrary to the common finding of decreased DMN activity during demanding, externally focused cognition, Crittenden et al. (2015) found core and MTL subnetworks to increase activity during the most demanding, between-domain switch trials (with the dmPFC subnetwork, if anything, showing the reverse). In all 3 subnetworks, furthermore, multivoxel pattern analysis (MVPA) showed distinct activity patterns for different trial types, in particular for the 3 different stimulus domains. Apparently, DMN activity can be seen not only in internally directed cognition, but in some aspects of external task switching when instruction cues call for retrieval and implementation of a new set of task rules.

Here we followed up this lead to DMN function. Given the unexpectedness of the Crittenden et al. (2015) results, our first aim was to replicate them using a new set of tasks. As in the previous study, participants switched between trials involving multiple rules and stimulus domains. To relate task switching activity to standard “rest” activity, we included cued rest trials in which participants simply rested, waiting for the next trial to begin. Importantly, these rests were short, of the order of the duration of a single trial, giving participants little time for explicit mind-wandering. Incorporation of rest trials, furthermore, allowed us to examine the switch back from rest to task (restart trials). To separate task preparation from execution, we introduced a delay between the colored cue instructing which task to perform next, and the imperative stimulus allowing the task to be executed. Following on from Crittenden et al. (2015), we assessed univariate switch and rest-related activity and task-related multivariate activity patterns in previously defined core, MTL and dmPFC subnetworks (Andrews-Hanna et al. 2010), as well as the typical task-positive MD network (Fedorenko et al. 2013). Our results are inconsistent with views of DMN function strictly linked to internal or self-directed cognition. In line with many others (Ranganath and Ritchey 2012), we propose that the DMN indeed encodes scene, episode or context, but assign this context encoding a direct role in implementation and control of current, externally focused cognition as well as internal thought processes.

## Materials and Methods

### Participants

A total of 28 participants (13 females), between 18 and 29 years old, were recruited through the Medical Research Council Cognition and Brian Sciences Unit volunteer panel. All participants were right handed, native English speakers, with normal or corrected to normal vision, and normal color vision. Ethics approval was granted from the Cambridge Psychology Research Ethics Committee. Four participants (3 females) were excluded from further analysis due to technical error (3) or participant noncompliance (1).

### Task

**Figure 1.**
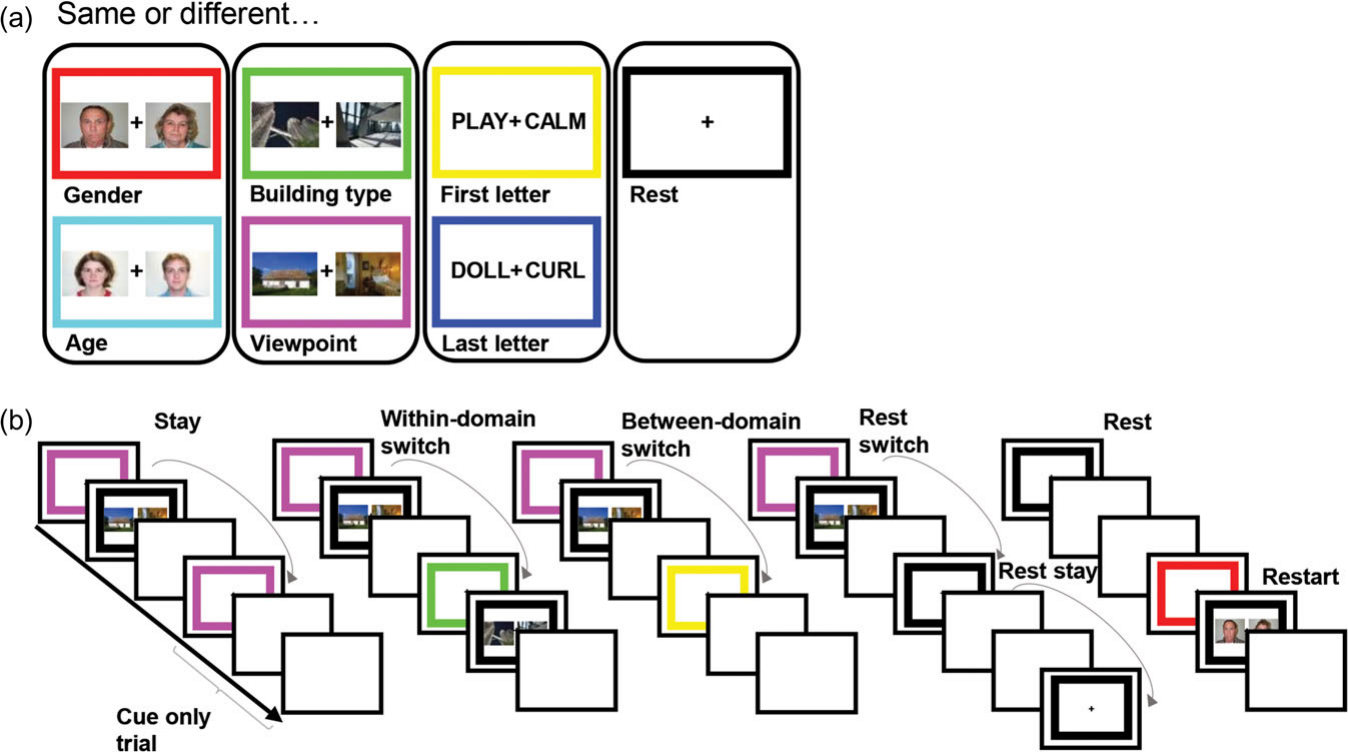
Task design. Participants were required to make same/different judgements on pairs of stimuli based on a task rule. Each task rule was cued by the frame color, learnt by participants in a training session prior to scanning. (*a*) Each of the 6 tasks and their associated frame color. There were 3 stimulus domains with 2 task rules associated with each. An additional black frame cued rest trials in which there was no upcoming task to complete. (*b*) Experimental design. Each trial consisted of a 2 s cue phase in which the colored frame specifying the task rule for the upcoming trial (or rest trial) was presented, followed by an execution phase (until response) or a 1.2 s delay, followed by a 1.75 s intertrial interval. “Cue-only trials” refer to task trials where there was no execution phase. The tasks were presented in a pseudorandom order creating 6 switch conditions: task stay trials (task trials preceded by the same task), within-domain switch (task trials preceded by same domain task trials), between-domain switch (task trials preceded by different domain task trials), rest switch (rest trials preceded by task trials), rest stay (rest trials preceded by rest trials), and restart (task trials preceded by rest trials).

Task events are illustrated in Figure 1. Participants were required to make same/different judgements on pairs of simultaneously presented stimuli based on a task rule. There were 3 stimulus domains with 2 task rules associated with each (male/female and old/young for face stimuli; skyscraper/cottage and inside/outside view for building stimuli; first letter and last letter for word stimuli). A further rest condition was added in which there was no task for participants to complete.

Trials of the 7 tasks (including rest) were presented in a pseudorandom order. This allowed for 6 switch conditions: rest switch (rest trials preceded by task trials), rest stay (rest trials preceded by rest trials), restart (task trials preceded by rest trials), between-domain switch (task trials preceded by different-domain task trials), within-domain switch (task trials preceded by same-domain task trials), and task stay trials (task trials preceded by the same task).

Each trial was split into 2 phases. In the 2 s cue phase, a colored frame was presented. Each color corresponded to a task or rest trial as represented in Figure 1. In the execution phase of task trials, 2 stimuli would appear and the colored frame would turn black. Participants were asked to make a “same” or “different” response, by left or right keypress, based on the task specified by the color of the frame in the cue period. There were equal numbers of “same” and “different” trials in each task and switch type. Performance was self-paced, with the imperative stimuli remaining until a key was pressed, and participants were asked to respond as quickly as possible without making mistakes. Which button corresponded to “same” and “different” was counterbalanced across participants. An intertrial interval (ITI) of 1.75 s followed each response. To improve isolation of brain activity associated with cues—the main focus of the study—33% of trials were catch trials, with no execution phase. Instead of an imperative stimulus, catch trials had an additional 1.2 s of ITI, matched to the average response time in a behavioral pilot study. This same additional ITI also followed the cue phase of rest trials.

The experiment consisted of 3 blocks of 217 trials each. Each block contained 36 task stay, 36 within-domain switch, 36 between-domain switch, 36 rest switch, 12 rest stay, and 24 restart trials. Of the task stay, within-domain switch and between-domain switch trials, 24 contained a task execution stage and 12 were catch trials. All restart trials were full trials, including task execution. There were equal numbers of each task type for each of the task switch conditions (task stay, within-domain switch, between-domain switch and restart). In addition to the above main trials, each block contained the first trial (switch type undefined), and 36 dummy trials (trials following catch trials). Dummy trials were all full trials, equally split between task types, and discarded from further analysis.

Stimuli were sourced from Wikimedia Commons and the Park Aging Mind Laborato-ry face database (Minear and Park 2004). Each stimulus was positioned either side of the fixation with 3.6° of visual angle from stimulus center to fixation. Each stimulus measured approximately 6.0 (width) × 4.5 (height) degrees of visual angle. The experiment was controlled using Psychophysics Toolbox for MATLAB (Brainard and Vision 1997).

### Training

Participants were carefully pretrained to ensure good learning of task rules. First, they were shown pairs of stimuli from each domain and asked to make same/different judgements according to each of the 6 task rules. Participants were then asked to learn the color of frame associated with each task rule using self-paced pen and paper memory tests. Participants were then introduced to rest trials and catch trials. To ensure fluid retrieval of task rule by frame color, a series of colored frames was presented on a monitor and participants were asked to name aloud the corresponding task rule. This portion of the training was complete when participants completed 2 cycles of frame colors without making a mistake. Finally, participants were given a practice block of the task. They were asked to use the cue period to prepare for the upcoming task. In the first 14 trials response feedback was given. The last 19 trials had no feedback and identical timings to the main task. Training lasted around 20 min, after which participants were moved into the scanner for their 3 task runs of approximately 20 min each. Before each run, participants were asked again to describe the rule associated with each cue color.

### Data Acquisition

Images were acquired using a 3 T Siemens Trim Trio magnetic resonance imaging (MRI) scanner, fitted with a 32-channel head coil. Functional MRI (fMRI) acquisitions used T2*-weighted multiband Echo-Planar Imaging (multiband acquisition factor 3 for 2.5 mm slices with no interslice gap, TR 1.1 s, TE 30 ms, flip angle 62°, voxel size 2 × 2 mm^2^). T1-weighted multiecho magnetization-prepared rapid gradient-echo (MPRAGE) images were also obtained (TR 2.25 s, TE 2.99 ms, flip angle 9°, voxel size 1 mm^3^).

### Preprocessing

Images were preprocessed using automaticanalysis (version 4) (Cusack et al. 2015) and SPM 12 (Wellcome Department of Cognitive Neurology, London, UK) for Matlab (Mathworks). The sequence of preprocessing stages involved spatial realignment of the raw EPIs, slice-time correction to the middle slice, coregistration of the functional EPI images to the structural T1-weighted image, and normalization to the Montreal Neurological Institute (MNI) template brain. For univariate analysis, functional images were then spatially smoothed using a Gaussian kernel of 10 mm full-width at half-maximum. No smoothing was used for multivariate analysis.

### Regions of Interest

**Figure 2.**
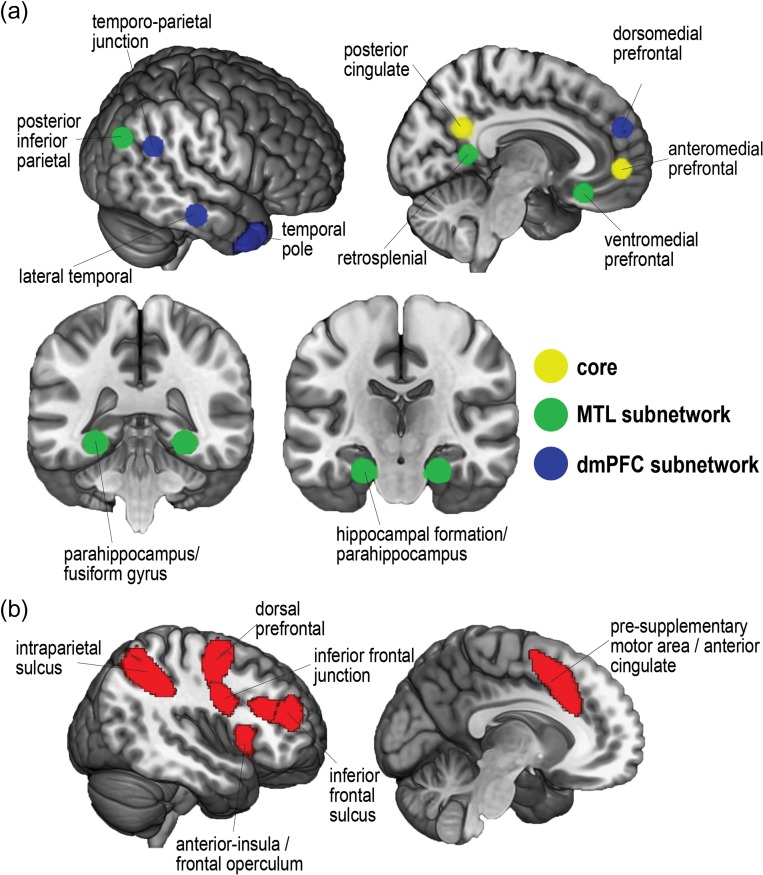
Regions of interest. (*a*) DMN ROIs from peak coordinates presented in Andrews-Hanna et al. (2010). (*b*) MD ROIs from Fedorenko et al. (2013).

To stay as close as possible to the DMN subnetworks defined by Andrews-Hanna et al. (2010), we generated DMN ROIs as 8 mm radius spheres around peak coordinates from that study. DMN ROIs are shown in Figure 2*a*. Due to the position of the bounding box, some voxels surrounding Andrews-Hanna et al. (2010) original peak temporal pole coordinates were not measured; to amend this, the temporal pole volumes as used in Crittenden et al. (2015) were each dilated in volume by 2 voxels. Frontoparietal MD ROIs were taken from Fedorenko et al. (2013). MD regions (Fig. 2*b*) included the posterior–anterior extent of the inferior frontal sulcus, dorsal prefrontal cortex, inferior frontal junction, anterior insula/frontal operculum, presupplementary motor area/dorsal anterior cingulate, and intraparietal sulcus. A template for these regions can be downloaded from http://imaging.mrc-cbu.cam.ac.uk/imaging/MDsystem. By using the version which separates each ROI, we were able to select only the frontoparietal ROIs.

### Univariate Analyses

Data for each participant were examined using the General Linear Model. Regressors were separately created for each combination of switch condition (task stay, within-domain switch, between-domain switch, restart, rest switch, rest stay, dummy) by task type (gender, age, building type, viewpoint, first letter, last letter) by task phase (cue, execution). Response type (same or different) was also separated for execution phase regressors. Incorrect trials were modeled separately and discarded. Dummy trials were also excluded from further analysis. Each regressor was modeled as a delta function convolved with the canonical hemodynamic response function, positioned at the onset of the cue periods and the middle of each execution period. Except for the restart condition, use of 33% trials with no execution phase meant that regressors could be well separated for cue and execution phases. Average contrast values were extracted for each ROI for each participant using the MarsBaR SPM toolbox (Brett et al. 2002), and contrast values were then averaged across ROIs for each DMN subnetwork. A similar analysis was also carried out for univariate activity in the MD network.

### Multivariate Analyses

MVPA was performed using the Decoding Toolbox (Christophel et al. 2012; Hebart et al. 2015). As with the univariate analysis, each regressor was modeled as a delta function convolved with the canonical hemodynamic response function, positioned at the onset of the cue period and the middle of each execution period. Incorrect trials were removed. MVPA then examined rule discrimination in patterns of cue phase activity, using the same ROIs as for univariate analysis. Prior to pattern analysis, beta values were *Z*-scored across tasks within each voxel of the ROI. Separate pairwise classifications were performed for each of the 15 possible task pairs (e.g., age vs. building type). Classification was carried out using a linear support vector machine (LIBSVM) (Fan et al. 2005) and a leave-one-run-out approach, with the classifier trained on data from 2 runs and tested on the third, and results averaged over the 3 possible left-out runs. Classification accuracy (CA) minus chance (50%) was generated for each classification pair, for each ROI and participant. The CA for each subnetwork was then computed from the average CA across ROIs in the subnetwork. Again, a similar analysis was also carried out for the MD network.

### Finite Impulse Response Model

To examine restart activity against a stable resting baseline, in a supplementary analysis we focused on occasions in which 4 rest trials appeared in a row, followed by restart. This analysis used a Finite Impulse Response model (FIR) model, where each trial was split into 4 parts (1.2375 s bins), extending from the onset of the first rest to 9.9 s after restart. We modeled the execution phases for each task switch type (task stay, within-domain switch, between-domain switch, and restart) as before, such that activation related to task execution was regressed out of the implicit baseline and FIR rest/restart estimates. Each execution regressor was modeled as a delta function convolved with the canonical hemodynamic response function, positioned in the middle of each execution period. The remaining implicit baseline contained intertrial periods as well as responses to all other cue events (i.e., stay, within-domain switch, between-domain switch, and remaining rest and restart cues). Average contrast values were extracted for each ROI for each participant using the MarsBaR SPM toolbox (Brett et al. 2002), and contrast values were then averaged across ROIs as before.

## Results

### Behavioral Switch Costs

Participants performed with an average of 95.9% correct responses (SD = 0.03). Paired-sample *t*-tests showed that responses were significantly faster for task stay trials (1217 ms) compared with within-domain switch trials (1373 ms, *t*[23] = 4.87, *P* < 0.01), between-domain switch trials (1348 ms, *t*[23] = 3.97 *P* < 0.01), and task restarts (1239 ms, *t*[23] = 4.22, *P* < 0.01). In contrast to the results of Crittenden et al. (2015), there was no significant difference between within- and between-domain switches. This is likely due to the introduction of a 2 s switch cue before the onset of the imperative stimulus making this design less sensitive to behavioral switch costs but allowed for estimation of switch-related activity independent of stimulus and execution effects.

### Increased DMN Activity on Rest Trials

**Figure 3.**
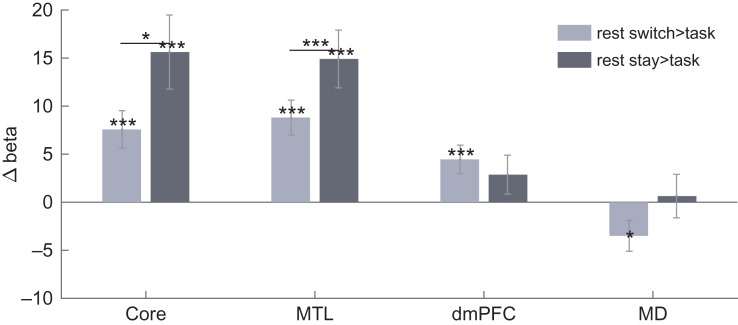
Contrasts of rest compared with task activity in each DMN subnetwork and the MD network. Significant (*P* < 0.05) increases in rest activity compared with task, as well as paired *t*-tests between contrasts within DMN subnetworks, are indicated with *P < 0.05, **P < 0.02, ***P < 0.01. Error bars show standard error of the mean across participants.

Given our interest in cognitive switching, our fMRI analyses focused largely on cue-related activity, with activity for task execution removed (see Materials and Methods). Our first analysis tested for the typical “task-negative” characteristic of the DMN, with stronger activity during rest compared with task. To this end, for each DMN subnetwork, we compared cue activity on rest and task trials, the latter defined as the mean of task stay, within-domain switch and between-domain switch trials. For all univariate analyses, average contrast values for each region of interest (ROI) were extracted, and contrast values were then averaged across ROIs within each subnetwork (see Materials and Methods). Figure 3 shows average contrast values for rest switch > task and rest stay > task. There were significant increases in activity during rest switch compared with task in all DMN subnetworks (core: *t*[23] = 3.88, *P* < 0.01; MTL: *t*[23] = 4.86, *P* < 0.01; dmPFC: *t*[23] = 3.00, *P* < 0.01), and for rest stay compared with task in the core and MTL subnetworks (core: *t*[23] = 4.05, *P* < 0.01; MTL: *t*[23] = 4.96, *P* < 0.01), but not in the dmPFC subnetwork (*t*[23] = 1.41, *P* > 0.05). Additional *t*-tests revealed increased activity in rest stay compared with rest switch trials only in core and MTL subnetworks (core: *t*[23] = 2.48, *P* < 0.05; MTL: *t*[23] = 3.94, *P* < 0.01; dmPFC: *t*[23] = 0.36, *P* > 0.05). To compare subnetworks, we ran a 2-way repeated measures ANOVA with factors contrast (rest stay > task stay, rest switch > task stay) and DMN subnetwork (core, MTL, dmPFC). While the effect of contrast was not significant (*F*[1,23] = 3.81, *P* > 0.05), significant main effects of subnetwork (*F*[2,46] = 15.86, *P* < 0.01) and a significant interaction effect were found (*F*[2,46] = 11.11, *P* < 0.01).

For comparison, Figure 3 also shows average contrast values for rest switch > task and rest stay > task in MD regions. MD regions showed significantly greater activity during task than switches to rest (*t*[23] = 2.18, *P* < 0.05), but showed no significant differences between rest stay compared with task (*t*[23] = 2.78, *P* > 0.02).

### Increased DMN Activity for Large Task Switches

**Figure 4.**
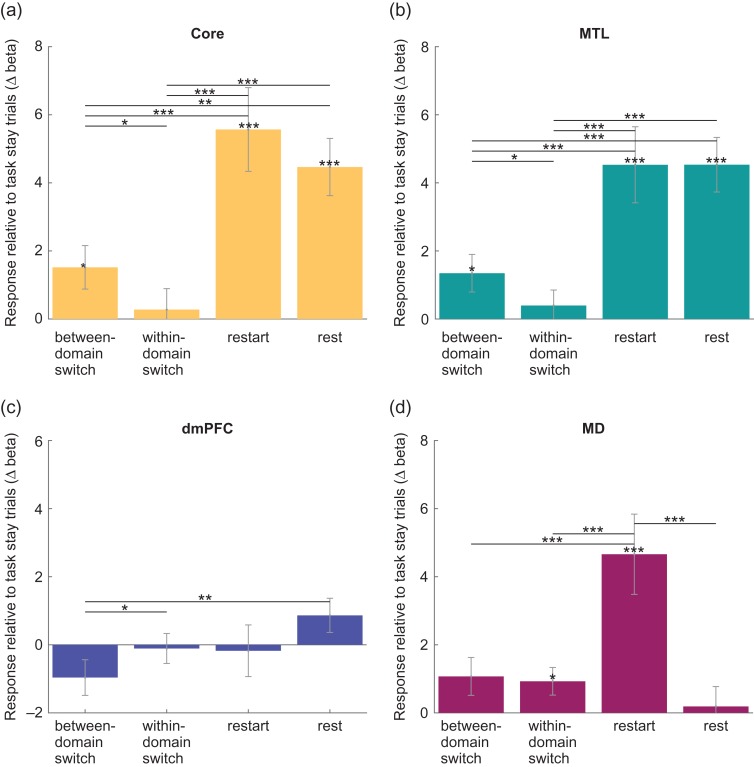
Contrasts of between-domain switch, within-domain switch, restart and rest compared with task stay trials for each DMN subnetwork and MD regions. ((*a*) Core DMN, (*b*) MTL DMN, (*c*) dmPFC DMN, (*d*) MD). Significant (*P* < 0.05) increases in activity compared with task stay, as well as paired t-tests between contrasts within (sub)networks, are indicated with *P < 0.05, **P < 0.02, ***P < 0.01. Error bars show standard error of the mean across participants.

Second, we aimed to replicate the results of Crittenden et al. (2015), showing increased activity in core and MTL subnetworks for between-domain switch trials. Figure 4*a*–*c* shows the effects of switch condition on cue period activity in each DMN subnetwork. Core and MTL DMN subnetworks showed increased activity for between-domain switches compared with both within-domain switches (core: *t*[23] = 2.17, *P* < 0.05; MTL: *t*[23] = 2.23, *P* < 0.05) and task stay trials (core: *t*[23] = 2.38, *P* < 0.05; MTL: *t*[23] = 2.44, *P* < 0.05). In line with trends reported by Crittenden et al. (2015), the dmPFC subnetwork showed the opposite effects of switch type, with decreased activity for between-domain switches compared with within-domain switches (*t*[23] = 2.20, *P* < 0.05). Within-domain switch trials were not significantly different from task stay trials.

In a supplementary analysis, no significant effects of switch type were found during the execution phase for core and MTL DMN subnetworks, while the dmPFC subnetwork showed decreased activity for between-domain switch trials compared with within-domain switch (*t*[23] = 2.54, *P* < 0.02) and task stay (*t*[23] = 3.07, *P* < 0.01).

### Large Increases in DMN Activity for Task Restarts

Finally we were interested in restart activity, that is, activity on trials where participants switched back from rest to task. For each DMN subnetwork, Figure 4*a*–*c* also shows the contrast values for restart > task stay, with rest > task stay (mean of rest stay and rest switch trials) added for comparison. For core and MTL, but not dmPFC, subnetworks, *t*-tests revealed increased activity during both restart (core: *t*[23] = 4.53, *P* < 0.01; MTL: *t*[23] = 4.05, *P* < 0.01; dmPFC: *t*[23] = 0.23, *P* > 0.05) and rest (core: *t*[23] = 5.31, *P* < 0.01; MTL: *t*[23] = 5.65, *P* < 0.01; dmPFC: *t*[23] = 1.72, *P* > 0.05) compared with task stay.

Our design allowed for separation of cue and execution-related activity in task stay, within-domain switch and between-domain switch trials. This was achieved by including cued task trials without an execution phase, reducing the covariance between the 2 task phases. However, all restart trials were full trials including an execution phase. As such, our design could not separate cue and execution components in this condition. To check our conclusions in relation to restart-related activity, we repeated analyses using beta values from whole trials (defined as the average beta values of both cue and execution phases of restart and task stay trials). These analyses similarly found increased activity for restart compared with task stay activity in core and MTL DMN subnetworks (core: *t*[23] = 5.07, *P* < 0.01; MTL: *t*[23] = 5.98, *P* < 0.01) and increased activity during rest compared with task stay in the core DMN subnetwork (*t*[23] = 5.62, *P* < 0.01). MTL rest activity was not greater than whole task stay trial activity. In the dmPFC DMN subnetwork, rest activity was greater than task stay (*t*[23] = 6.07, *P* < 0.01) but restart was not.

### Component ROIs Within Each Subnetwork

Supplementary Figures S1–S3 show the above contrasts plotted for individual ROIs in each DMN subnetwork. Core DMN subnetwork regions showed similar patterns of response across rest and restart contrasts, although the task switch response was driven by PPC and absent in aMPFC. Within the MTL subnetwork, all regions responded strongly to rest, the task switch response was driven by Rsp and PHC, and the restart response was seen everywhere except the posterior intraparietal lobe. The dmPFC subnetwork show less convergence across individual ROIs, consistent with its weak effects overall.

### MD Activity Across Trial Types

For comparison with the DMN data (Fig. 4*a*–*c*), Figure 4d shows contrasts of different trial types with task stay for the MD network. As in core and MTL subnetworks, *t*-tests showed greater MD activity for restart (*t*[23] = 3.95, *P* < 0.01). MD activity was also greater in within-domain switch trials (*t*[23] = 2.29, *P* > 0.05) compared with task stay trials. Between-domain switch activity was not significantly different from task stay activity although there was also no difference between responses to within-domain switches and between-domain switches. In contrast to Core and MTL DMN subnetworks, the response on rest trials was not significantly greater than on task stay trials. Again, a supplementary analysis showed no significant effects of switch type during task execution.

To compare effects of switch magnitude in DMN and MD, we ran 2 two-way repeated measures ANOVAs, with factors contrast (between-domain switch > task stay, within-domain switch > task stay) and network (core, MD in the first analysis; MTL, MD in the second). The ANOVA comparing core DMN with MD activity found no significant main effects of contrast or network, but a significant interaction (*F*[1,23] = 5.05, *P* < 0.05). Results were similar in the ANOVA comparing MTL DMN with MD, with no significant main effects of contrast or network, but a significant interaction (*F*[1,23] = 6.56, *P* < 0.02). The results show that the effect of switch magnitude was greater in DMN subnetworks than in MD.

### Increased Activity at Task Restart is Distinct From Prolonged Rest Activity

A potential concern over restart activity is that, in part, it might reflect carry-over from the preceding rest, either because of sustained neural activity, or a prolonged haemodynamic response. To examine activity at restart in more detail, and in particular to compare restart against a stable resting baseline, we selected instances in which at least 4 rest trials occurred in a row (data available for all 24 participants; mean of 3.88 instances per participant). Using a Finite Impulse Response model (see Materials and Methods), we estimated activity in 1.2375 s time bins (4 bins/trial) across the run of 4 rests and into the following restart.

**Figure 5.**
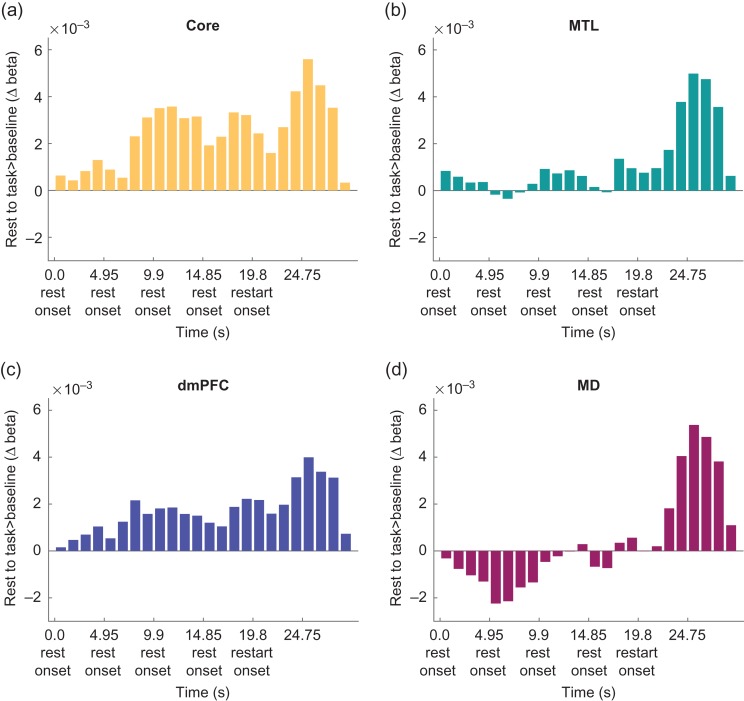
Finite impulse response beta activity estimates in 1.1 s time bins during repeated rest trials before task restart for each DMN subnetwork and the MD network. ((*a*) Core DMN, (*b*) MTL DMN, (*c*) dmPFC DMN, (*d*) MD). Restart onset occurs at bin 19.

The results are shown in Figure 5. Across all 3 DMN subnetworks, the results suggest a peak of activity following restart. At least after long rests, in other words, a restart drives stronger DMN activity than the rest trials themselves. To compare restart with the immediately preceding rest, we averaged across the 3 bins corresponding to 3.7125–7.425 s after restart (bins 20–22) and the 3 corresponding bins for the final rest (bins 16-18). A two-way repeated measures ANOVA with the factors DMN subnetwork (core, MTL and dmPFC) and trial (restart, rest) showed a main effect of trial that fell just short of significance (*F*[1,23] = 3.86, *P* = 0.062), along with a significant interaction (*F*[1,23] = 4.17, *P* < 0.05). The restart effect appeared largest in the MTL subnetwork. We also found significant increases in response to restart compared with rest in the MD network (*t*[23] = 3.46, *P* < 0.01).

### Individual Voxels in DMN and MD Regions Show Sensitivity to Both Rest and Between-Domain Task Switches

To investigate whether voxels within the same regions were sensitive to rest as well as to the between-domain task switches, for each participant in each ROI we calculated the proportion of voxels showing above threshold responses to rest > task, between-domain switch > task stay, and to both rest > task and between-domain switch > task stay contrasts, at the threshold value of *P* < 0.05, uncorrected. All (sub)network regions showed some voxels sensitive to both rest and between-domain switches with a large proportion of between-domain switch sensitive voxels also sensitive to rest (core: 18.3%, MTL: 26.9%, dmPFC: 11.7%, MD: 10.8%).

### DMN and MD Activity Patterns Distinguish Task Domains

Multivariate analyses were also carried out to establish whether DMN cue period activity could distinguish between different task types. For each task pair (e.g., age vs. building type), a support vector machine (LIBSVM; Fan et al. 2005) was trained to discriminate the 2 tasks, based on voxelwise activity patterns in each DMN ROI separately (see Materials and Methods). CA was assessed using a leave-one-run-out procedure, and expressed as accuracy minus chance (50%). The CA for each subnetwork was then computed from the average CA of each ROI in the subnetwork.

**Figure 6.**
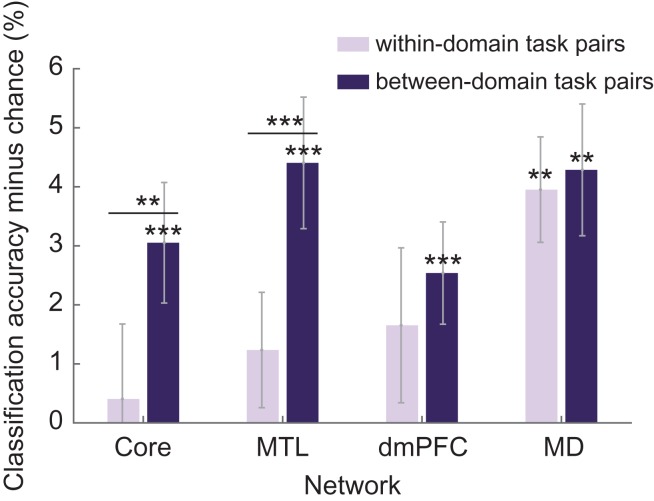
Average classification accuracies minus chance for within-domain (light bars) and between-domain (dark bars) task pairs for each DMN subnetwork and the MD network. Significant classification accuracy above chance (*P* < 0.05), as well as significant paired *t*-tests between within-domain task pairs and between-domain task pairs, are indicated with *P < 0.05, **P < 0.02, ***P < 0.01. Error bars show standard error of the mean across participants.

Separately for within- and between-domain task pairs, Figure 6 shows mean values of CA minus chance for each DMN subnetwork, along with results of a similar analysis for the MD network. Although mean classification accuracies were low, *T*-tests showed CA significantly above chance for between-domain task pairs in all DMN subnetworks as well as the MD network (core: *t*[23] = 2.99, *P* < 0.01; MTL: *t*[23] = 3.95, *P* < 0.01; dmPFC: *t*[23] = 2.93, *P* < 0.01; MD: *t*[23] = 3.84, *P* < 0.01). Only in MD regions was CA of within-domain task pairs significantly above chance (*t*[23] = 4.42, *P* < 0.01). Paired *t*-tests revealed a significant increase in CA for between-domain task pairs compared with within-domain task pairs in core and MTL subnetworks (core: *t*[23] = 2.59, *P* < 0.02; MTL: *t*[23] = 3.38, *P* < 0.01). A two-way repeated measures ANOVA was also carried out with the factors network (DMN, MD) and task pair similarity (within-domain, between-domain). Data were averaged over core, MTL and dmPFC subnetworks to obtain DMN values. Significant main effects of network (*F*[1,23] = 8.86, *P* < 0.01) and similarity (*F*[1,23] = 4.86, *P* < 0.05) were found, as well as a significant interaction (*F*[1,23] = 4.85, *P* < 0.05).

## Discussion

In this study we aimed to extend a prior suggestion (Crittenden et al. 2015) that regions of the DMN can play a role in externally directed task switching. First, we wished to replicate DMN activation during large task switches. Second, we wished to link such switch-related activity to the typical profile of DMN activation during rest. Third, our design allowed us to examine the cognitive transition from rest to task.

Our findings confirm the role of the DMN in cognitive transitions. In line with Crittenden et al. (2015), we found increased activity in core and MTL subnetworks for a large change of task domain but not for a within-domain task change. In line with prototypical findings, we also found increased DMN activity on rest trials compared with task. Perhaps most striking, core and MTL subnetworks were also strongly active during the transition back from rest to task, with further analyses showing some overlap between voxels most active for rest and those showing task switch effects.

In the Crittenden et al. (2015) study, it was left open whether DMN activity during a switch of task domain was caused by relaxation of the previous task set, or establishment of the next. Our introduction of rest trials indicates DMN activity for both. Activity was strong both when a previous cognitive focus was relaxed (switch from task to rest), and when a new one was established (switch from rest to the next task).

Taken together, our results are difficult to explain using theories of DMN function strictly linked to internal or self-directed cognition. Inconsistent with theories suggesting a role for the DMN in processes directed away from the current external environment (e.g., self-projection, Buckner and Carroll 2007), we see DMN switch-related activity changes in the context of an external task. Furthermore, large DMN activity increases during short rest trials seem unlikely to be caused by immediate spontaneous mind-wandering, autobiographical memory recall or self-related cognition. Substantial activity at task restart is directly inconsistent with these theories. These results call for a conceptualization of DMN function that simultaneously addresses activity during rest, including brief task pauses, during typical “internally directed” cognition, and during large external task switches.

As discussed earlier, many results in the literature suggest that DMN regions represent broad features of a current scene, episode or context, including spatial surroundings (Hassabis and Maguire 2007, 2009; Howard et al. 2014), time (Addis et al. 2007; Andrews-Hanna et al. 2010), social aspects of self and others (Frith and Frith 2003; D’Argembeau et al. 2005), etc. This has been called a “situation model” by Ranganath and Ritchey (2012). This context encoding, we suggest, can indeed be important in imagining contexts different from the current moment, but also plays a role in implementation and control of current cognition. Various possible roles might be proposed for context representations. For example, current spatial, temporal and social context could constrain what kind of behavior is currently possible, desirable or permitted. When new behavior is assembled, it makes sense that there should be reference to the options permitted by the current broad context. As another possibility, the components of current behavior must always be bound together and supported against potentially competing alternatives. Temporary association with the current, relatively stable context could be one way to achieve this.

We suggest that, as cognition unfolds, there is a constant waxing and waning of the relative prominence of context representation, and hence of DMN activity. Context representation is strong at rest, perhaps because little else competes for cognitive resources. Sometimes this represented context may be internally constructed through engagement in self-generated thought, but equally, representation of current surroundings may simply be strengthened. Complementarily, as we progressively become embedded in the operations of a focused task, context representation may recede, perhaps because they are not relevant and are suppressed through selective attention. This weakening of context representations could correspond to the experience of “losing ourselves” in ongoing activity, and to well-known anticorrelations between task-negative and task-positive regions (Fox, Snyder, et al. 2005; Kelly et al. 2008). With a major switch to a new task, however, we suggest that context representation is reawakened, allowing rereference of the new task to current surroundings. Again, this might correspond to the common experience of “becoming aware of our surroundings” when a current cognitive focus is interrupted.

Our proposal is consistent with several findings implying overlap between representations of current and internally generated scenes. Firstly, work on the perceptuo-mnemonic hypothesis (Buckley et al. 2001) suggests that MTL regions are part of a perceptual processing hierarchy as well as being important for memory. DMN regions (hippocampus and parahippocampus) implicated in memory for scenes are also necessary for the perceptual processing of scenes (Lee et al. 2005, 2006). MVPA studies have found that spatial and temporal patterns of activity in multiple DMN regions represent scene specific contextual information when both viewing and later recalling the same episode of a TV program (Baldassano et al. 2016; Chen et al. 2017). Additional MVPA studies suggest that information represented in DMN regions is not limited to the visuospatial domain. Baldassano et al. (2016) found similar patterns of DMN activity for audio-descriptions of the same television scenes, and, most relevant to our findings, research by Schuck et al. (2016) found DMN activity could decode between different nonspatial task contexts (house or face judgements). Research on grid cells further suggests that DMN regions can provide contextual structure for multiple domains of stimuli. Although grid cells are strongly implicated in representing the spatial structure of the environment (Doeller et al. 2010; Jacobs et al. 2013; Kraus et al. 2015), Constantinescu et al. (2016) recently found that grid cells in the entorhinal cortex and ventromedial prefrontal cortex could also represent conceptual knowledge structures, such as the neck:legs ratio in an artificial “bird space”.

At cognitive transitions, new behavioral rules must be implemented. In part, these rules are retrieved from memory, as in the present study, and as DMN activity is common during episodic retrieval, might activity at transitions also reflect a retrieval demand? Though we propose that context representations are important in retrieving or establishing new rules, DMN activity even for short rest trials suggests that retrieval itself is not a necessary condition for strengthening of DMN representations. There are also retrieval tasks, such as N-back, which show no DMN activity (Owen et al. 2005). Though some aspects of “retrieval” are evidently linked to the DMN, more is needed to establish which types or aspects of retrieval are most relevant.

Intriguingly, in our data, DMN regions showed some task-related patterns of activity similar to those of MD regions, with strong increases at between-domain switches and restart. These results match occasional previous reports that DMN and MD activity are not necessarily anticorrelated (Christoff et al. 2009; Spreng et al. 2010; Gerlach et al. 2011; Dixon et al. 2017; Margulies and Smallwood 2017). In line with studies implicating MD activity in task set implementation (Dosenbach et al. 2006; Duncan 2010, 2013; Crittenden et al. 2016), we suggest that during task restarts DMN and MD regions work together. During task restarts, DMN regions could be responsible for representation and assessment of the broad cognitive context, enabling the MD network to implement a specific task set. During large switches to a different task domain in particular, the broad task context may be briefly re-activated in order to double-check the task constraints relating to this large switch. These suggestions match the broad proposal that DMN and MD systems play complementary roles in the organization of complex, goal-directed behavior (Margulies and Smallwood 2017). Despite these similarities, DMN and MD networks also showed important differences. While DMN showed increased activity only for large, between-domain task switches, even small, within-domain switches recruited MD regions. Our MVPA results were consistent with this distinction, indicating only relatively coarse task representations in the DMN, while MD activity was able distinguish on a finer scale between all 6 tasks.

As task restart trials always followed rest trials, one possibility is that high DMN activity at restart could reflect slow decay of neural activity, or simply a prolonged haemodynamic response, following rest. However, the results from our FIR model for long rest runs tell against this possibility. In all DMN subnetworks, the data show that, following restart, activity increased beyond the level established during the preceding rest (Fig. 5). These results show that restart itself recruits strong DMN activity. This finding is also supported by Fox, Snyder, Barch, et al. (2005) who found transient task onset activity in DMN regions including the posterior cingulate, precuneus and temporoparietal junction across 4 different tasks. Rest activity also showed a transient component, with a peak at rest onset followed by decay as rest continued. Our data, as well as Fox, Snyder, Barch, et al. (2005), strongly link DMN activity to cognitive transitions from rest to task as well as task to rest.

In line with the findings of Andrews-Hanna et al. (2010) and Crittenden et al. (2015), our results show substantial differences between DMN subnetworks. While activity was very similar for core and MTL subnetworks, the dmPFC network behaved quite differently, with reduced activity on switch trials, and only modest increase for rest trials. Like core and MTL subnetworks, however, the dmPFC subnetwork did show MVPA encoding of task domain. Whilst being consistent with Crittenden et al. (2015), the activity pattern in the dmPFC subnetwork is difficult to interpret. Andrews-Hanna et al. (2010) suggested that dmPFC subnetwork regions might be important for self-referential and social cognitive processes, which have been found to share considerable neural overlap (Saxe et al. 2006; Lombardo et al. 2010). One possibility is that, in a study like ours, reinstatement of social context has little involvement in cognitive transitions; potentially it is more important in everyday events, in which social context may be richer and more variable.

In summary, we have implicated the DMN in cognitive transitions, not just in internally focused tasks but externally focused tasks also. Just as it encodes internally generated cognitive scenes, episodes or contexts, we suggest the DMN also encodes current, external context. Along with DMN activity, context encoding may weaken as similar cognitive operations are repeated, but reappear when major cognitive transitions call for contextual rereference. The DMN, we suggest, is not involved simply in mind-wandering, imagination, or recollection; its contextual representations are important in shaping both internally and externally directed cognition.

## Supplementary Material

Supplementary Data

## Footnotes

*Conflict of Interest*: None declared.

## References

[bhy167C1] Addis DR, Wong AT, Schacter DL 2007 Remembering the past and imagining the future: common and distinct neural substrates during event construction and elaboration. Neuropsychologia. 45:1363–1377. 17126370 10.1016/j.neuropsychologia.2006.10.016 PMC1894691

[bhy167C2] Amodio DM, Frith CD 2006 Meeting of minds: the medial frontal cortex and social cognition. Nat Rev Neurosci. 7:268–277. 16552413 10.1038/nrn1884

[bhy167C3] Andrews-Hanna JR, Reidler JS, Sepulcre J, Poulin R, Buckner RL 2010 Functional-anatomic fractionation of the brain’s default network. Neuron. 65:550–562. 20188659 10.1016/j.neuron.2010.02.005 PMC2848443

[bhy167C4] Bachevalier J, Nemanic S 2008 Memory for spatial location and object‐place associations are differently processed by the hippocampal formation, parahippocampal areas TH/TF and perirhinal cortex. Hippocampus. 18:64–80. 17924520 10.1002/hipo.20369

[bhy167C5] Balaguer J, Spiers H, Hassabis D, Summerfield C 2016 Neural mechanisms of hierarchical planning in a virtual subway network. Neuron. 90:893–903. 27196978 10.1016/j.neuron.2016.03.037 PMC4882377

[bhy167C6] Baldassano C, Chen J, Zadbood A, Pillow JW, Hasson U, Norman KA 2016 Discovering event structure in continuous narrative perception and memory. bioRxiv. 1:081018. 10.1016/j.neuron.2017.06.041 PMC5558154 28772125

[bhy167C7] Bar M 2007 The proactive brain: using analogies and associations to generate predictions. Trends Cogn Sci. 11:280–289. 17548232 10.1016/j.tics.2007.05.005

[bhy167C8] Bar M 2009 The proactive brain: memory for predictions. Philos Trans R Soc B Biol Sci. 364:1235–1243. 10.1098/rstb.2008.0310 PMC2666710 19528004

[bhy167C9] Bird CM, Burgess N 2008 The hippocampus and memory: insights from spatial processing. Nat Rev Neurosci. 9:182–194. 18270514 10.1038/nrn2335

[bhy167C10] Brainard DH, Vision S 1997 The psychophysics toolbox. Spat Vis. 10:433–436. 9176952

[bhy167C11] Brett M, Anton JL, Valabregue R, Poline JB 2002 Region of interest analysis using the MarsBar toolbox for SPM 99. Neuroimage. 16:S497.

[bhy167C12] Buckley MJ, Booth MC, Rolls ET, Gaffan D 2001 Selective perceptual impairments after perirhinal cortex ablation. J Neurosci. 21:9824–9836. 11739590 10.1523/JNEUROSCI.21-24-09824.2001 PMC6763048

[bhy167C13] Buckner RL, Carroll DC 2007 Self-projection and the brain. Trends Cogn Sci. 11:49–57. 17188554 10.1016/j.tics.2006.11.004

[bhy167C14] Burwell RD, Saddoris MP, Bucci DJ, Wiig KA 2004 Corticohippocampal contributions to spatial and contextual learning. J Neurosci. 24:3826–3836. 15084664 10.1523/JNEUROSCI.0410-04.2004 PMC6729354

[bhy167C16] Chen J, Leong YC, Honey CJ, Yong CH, Norman KA, Hasson U 2017 Shared memories reveal shared structure in neural activity across individuals. Nat Neurosci. 20:115–125. 27918531 10.1038/nn.4450 PMC5191958

[bhy167C17] Christoff K, Gordon AM, Smallwood J, Smith R, Schooler JW 2009 Experience sampling during fMRI reveals default network and executive system contributions to mind wandering. Proc Natl Acad Sci U S A. 106:8719–8724. 19433790 10.1073/pnas.0900234106 PMC2689035

[bhy167C18] Christophel TB, Hebart MN, Haynes JD 2012 Decoding the contents of visual short-term memory from human visual and parietal cortex. Journal of Neuroscience. 32:12983–12989. 22993415 10.1523/JNEUROSCI.0184-12.2012 PMC6621473

[bhy167C19] Constantinescu AO, O’Reilly JX, Behrens TE 2016 Organizing conceptual knowledge in humans with a gridlike code. Science. 352:1464–1468. 27313047 10.1126/science.aaf0941 PMC5248972

[bhy167C20] Corbetta M, Shulman GL 2002 Control of goal-directed and stimulus-driven attention in the brain. Nat Rev Neurosci. 3:201–215. 11994752 10.1038/nrn755

[bhy167C21] Crittenden BM, Mitchell DJ, Duncan J 2015 Recruitment of the default mode network during a demanding act of executive control. Elife. 4:e06481. 25866927 10.7554/eLife.06481 PMC4427863

[bhy167C22] Crittenden BM, Mitchell DJ, Duncan J 2016 Task encoding across the multiple demand cortex is consistent with a frontoparietal and cingulo-opercular dual networks distinction. J Neurosci. 36:6147–6155. 27277793 10.1523/JNEUROSCI.4590-15.2016 PMC4899522

[bhy167C23] Cusack R, Vicente-Grabovetsky A, Mitchell DJ, Wild CJ, Auer T, Linke AC, Peelle JE 2015 Automatic analysis (aa): efficient neuroimaging workflows and parallel processing using Matlab and XML. Front Neuroinform. 8:90. 25642185 10.3389/fninf.2014.00090 PMC4295539

[bhy167C24] Diana RA, Yonelinas AP, Ranganath C 2007 Imaging recollection and familiarity in the medial temporal lobe: a three-component model. Trends Cogn Sci. 11:379–386. 17707683 10.1016/j.tics.2007.08.001

[bhy167C25] Dixon ML, Andrews-Hanna JR, Spreng RN, Irving ZC, Mills C, Girn M, Christoff K 2017 Interactions between the default network and dorsal attention network vary across default subsystems, time, and cognitive states. Neuroimage. 147:632–649. 28040543 10.1016/j.neuroimage.2016.12.073

[bhy167C26] Doeller CF, Barry C, Burgess N 2010 Evidence for grid cells in a human memory network. Nature. 463:657–661. 20090680 10.1038/nature08704 PMC3173857

[bhy167C27] Dosenbach NU, Visscher KM, Palmer ED, Miezin FM, Wenger KK, Kang HC, Burgund ED, Grimes AL, Schlaggar BL, Petersen SE 2006 A core system for the implementation of task sets. Neuron. 50:799–812. 16731517 10.1016/j.neuron.2006.04.031 PMC3621133

[bhy167C28] Duncan J 2010 The multiple-demand (MD) system of the primate brain: mental programs for intelligent behaviour. Trends Cogn Sci. 14:172–179. 20171926 10.1016/j.tics.2010.01.004

[bhy167C29] Duncan J 2013 The structure of cognition: attentional episodes in mind and brain. Neuron. 80:35–50. 24094101 10.1016/j.neuron.2013.09.015 PMC3791406

[bhy167C30] Duncan J, Owen AM 2000 Common regions of the human frontal lobe recruited by diverse cognitive demands. Trends Neurosci. 23:475–483. 11006464 10.1016/s0166-2236(00)01633-7

[bhy167C31] D’Argembeau A, Collette F, Van der Linden M, Laureys S, Del Fiore G, Degueldre C, Luxen A, Salmon E 2005 Self-referential reflective activity and its relationship with rest: a PET study. Neuroimage. 25:616–624. 15784441 10.1016/j.neuroimage.2004.11.048

[bhy167C32] Fan RE, Chen PH, Lin CJ 2005 Working set selection using second order information for training support vector machines. J Mach Learn Res. 6:1889–1918.

[bhy167C33] Fedorenko E, Duncan J, Kanwisher N 2013 Broad domain generality in focal regions of frontal and parietal cortex. Proc Natl Acad Sci U S A. 110:16616–16621. 24062451 10.1073/pnas.1315235110 PMC3799302

[bhy167C34] Fox MD, Snyder AZ, Barch DM, Gusnard DA, Raichle ME 2005b. Transient BOLD responses at block transitions. Neuroimage. 28(4):956–966. 16043368 10.1016/j.neuroimage.2005.06.025

[bhy167C35] Fox MD, Snyder AZ, Vincent JL, Corbetta M, Van Essen DC, Raichle ME 2005a. The human brain is intrinsically organized into dynamic, anticorrelated functional networks. Proc Natl Acad Sci U S A. 102:9673–9678. 15976020 10.1073/pnas.0504136102 PMC1157105

[bhy167C500] Fransson P, Marrelec G 2008 The precuneus/posterior cingulate cortex plays a pivotal role in the default mode network: Evidence from a partial correlation network analysis. Neuroimage. 42:1178–1184. 18598773 10.1016/j.neuroimage.2008.05.059

[bhy167C36] Frith U, Frith CD 2003 Development and neurophysiology of mentalizing. Philos Trans R Soc Lond B Biol Sci. 358:459–473. 12689373 10.1098/rstb.2002.1218 PMC1693139

[bhy167C37] Gerlach KD, Spreng RN, Gilmore AW, Schacter DL 2011 Solving future problems: default network and executive activity associated with goal-directed mental simulations. Neuroimage. 55:1816–1824. 21256228 10.1016/j.neuroimage.2011.01.030 PMC3855008

[bhy167C38] Greicius MD, Krasnow B, Reiss AL, Menon V 2003 Functional connectivity in the resting brain: a network analysis of the default mode hypothesis. Proc Natl Acad Sci. 100:253–258. 12506194 10.1073/pnas.0135058100 PMC140943

[bhy167C39] Hassabis D, Maguire EA 2007 Deconstructing episodic memory with construction. Trends Cogn Sci. 11:299–306. 17548229 10.1016/j.tics.2007.05.001

[bhy167C40] Hassabis D, Maguire EA 2009 The construction system of the brain. Philos Trans R Soc B Biol Sci. 364:1263–1271. 10.1098/rstb.2008.0296 PMC2666702 19528007

[bhy167C41] Hebart MN, Görgen K, Haynes JD 2015 The Decoding Toolbox (TDT): a versatile software package for multivariate analyses of functional imaging data. Front Neuroinform. 8:88. 25610393 10.3389/fninf.2014.00088 PMC4285115

[bhy167C42] Howard LR, Javadi AH, Yu Y, Mill RD, Morrison LC, Knight R, Loftus MM, Staskute L, Spiers HJ 2014 The hippocampus and entorhinal cortex encode the path and Euclidean distances to goals during navigation. Curr Biol. 24:1331–1340. 24909328 10.1016/j.cub.2014.05.001 PMC4062938

[bhy167C43] Jacobs J, Weidemann CT, Miller JF, Solway A, Burke JF, Wei XX, Suthana N, Sperling MR, Sharan AD, Fried I, et al 2013 Direct recordings of grid-like neuronal activity in human spatial navigation. Nat Neurosci. 16:1188–1190. 23912946 10.1038/nn.3466 PMC3767317

[bhy167C44] Johnson SC, Baxter LC, Wilder LS, Pipe JG, Heiserman JE, Prigatano GP 2002 Neural correlates of self‐reflection. Brain. 125:1808–1814. 12135971 10.1093/brain/awf181

[bhy167C45] Kelly AC, Uddin LQ, Biswal BB, Castellanos FX, Milham MP 2008 Competition between functional brain networks mediates behavioral variability. Neuroimage. 39:527–537. 17919929 10.1016/j.neuroimage.2007.08.008

[bhy167C46] Konishi M, McLaren DG, Engen H, Smallwood J 2015 Shaped by the past: the default mode network supports cognition that is independent of immediate perceptual input. PLoS One. 10:e0132209. 26125559 10.1371/journal.pone.0132209 PMC4488375

[bhy167C47] Kraus BJ, Brandon MP, Robinson RJ, Connerney MA, Hasselmo ME, Eichenbaum H 2015 During running in place, grid cells integrate elapsed time and distance run. Neuron. 88:578–589. 26539893 10.1016/j.neuron.2015.09.031 PMC4635558

[bhy167C48] Kumaran D, Maguire EA 2005 The human hippocampus: cognitive maps or relational memory? J Neurosci. 25:7254–7259. 16079407 10.1523/JNEUROSCI.1103-05.2005 PMC6725222

[bhy167C49] Lee AC, Buckley MJ, Gaffan D, Emery T, Hodges JR, Graham KS 2006 Differentiating the roles of the hippocampus and perirhinal cortex in processes beyond long-term declarative memory: a double dissociation in dementia. J Neurosci. 26:5198–5203. 16687511 10.1523/JNEUROSCI.3157-05.2006 PMC6674247

[bhy167C50] Lee AC, Bussey TJ, Murray EA, Saksida LM, Epstein RA, Kapur N, Hodges JR, Graham KS 2005 Perceptual deficits in amnesia: challenging the medial temporal lobe ‘mnemonic’view. Neuropsychologia. 43:1–11. 15488899 10.1016/j.neuropsychologia.2004.07.017

[bhy167C51] Lombardo MV, Chakrabarti B, Bullmore ET, Wheelwright SJ, Sadek SA, Suckling J, MRC Aims Consortium, Baron-Cohen S 2010 Shared neural circuits for mentalizing about the self and others. J Cogn Neurosci. 22:1623–1635. 19580380 10.1162/jocn.2009.21287

[bhy167C52] Margulies DS, Smallwood J 2017 Converging evidence for the role of transmodal cortex in cognition. Proc Natl Acad Sci U S A. 114:12641–12643. 29142008 10.1073/pnas.1717374114 PMC5715795

[bhy167C54] Mason MF, Norton MI, Van Horn JD, Wegner DM, Grafton ST, Macrae CN 2007 Wandering minds: the default network and stimulus-independent thought. Science. 315:393–395. 17234951 10.1126/science.1131295 PMC1821121

[bhy167C55] Minear M, Park DC 2004 A lifespan database of adult facial stimuli. Behav Res Methods Instrum Comput. 36:630–633. 15641408 10.3758/bf03206543

[bhy167C56] Owen AM, McMillan KM, Laird AR, Bullmore E 2005 N‐back working memory paradigm: a meta‐analysis of normative functional neuroimaging studies. Hum Brain Mapp. 25:46–59. 15846822 10.1002/hbm.20131 PMC6871745

[bhy167C57] Ranganath C, Ritchey M 2012 Two cortical systems for memory-guided behaviour. Nat Rev Neurosci. 13:713–726. 22992647 10.1038/nrn3338

[bhy167C58] Saxe R, Moran JM, Scholz J, Gabrieli J 2006 Overlapping and non-overlapping brain regions for theory of mind and self reflection in individual subjects. Soc Cogn Affect Neurosci. 1:229–234. 18985110 10.1093/scan/nsl034 PMC2555418

[bhy167C59] Schacter DL, Addis DR, Buckner RL 2007 Remembering the past to imagine the future: the prospective brain. Nat Rev Neurosci. 8:657–661. 17700624 10.1038/nrn2213

[bhy167C60] Schuck NW, Cai MB, Wilson RC, Niv Y 2016 Human orbitofrontal cortex represents a cognitive map of state space. Neuron. 91:1402–1412. 27657452 10.1016/j.neuron.2016.08.019 PMC5044873

[bhy167C61] Shulman GL, Fiez JA, Corbetta M, Buckner RL, Miezin FM, Raichle ME, Petersen SE 1997 Common blood flow changes across visual tasks: II. Decreases in cerebral cortex. J Cogn Neurosci. 9:648–663. 23965122 10.1162/jocn.1997.9.5.648

[bhy167C62] Smallwood J, Tipper C, Brown K, Baird B, Engen H, Michaels JR, Grafton S, Schooler JW 2013 Escaping the here and now: evidence for a role of the default mode network in perceptually decoupled thought. Neuroimage. 69:120–125. 23261640 10.1016/j.neuroimage.2012.12.012

[bhy167C63] Spiers HJ, Maguire EA 2007 Decoding human brain activity during real-world experiences. Trends Cogn Sci. 11:356–365. 17618161 10.1016/j.tics.2007.06.002

[bhy167C64] Spreng RN, Mar RA, Kim AS 2009 The common neural basis of autobiographical memory, prospection, navigation, theory of mind, and the default mode: a quantitative meta-analysis. J Cogn Neurosci. 21:489–510. 18510452 10.1162/jocn.2008.21029

[bhy167C65] Spreng RN, Stevens WD, Chamberlain JP, Gilmore AW, Schacter DL 2010 Default network activity, coupled with the frontoparietal control network, supports goal-directed cognition. Neuroimage. 53:303–317. 20600998 10.1016/j.neuroimage.2010.06.016 PMC2914129

[bhy167C66] Vann SD, Aggleton JP 2002 Extensive cytotoxic lesions of the rat retrosplenial cortex reveal consistent deficits on tasks that tax allocentric spatial memory. Behav Neurosci. 116:85. 11895186

[bhy167C67] Vilberg KL, Rugg MD 2012 The neural correlates of recollection: transient versus sustained fMRI effects. J Neurosci. 32:15679–15687. 23136408 10.1523/JNEUROSCI.3065-12.2012 PMC3509997

