# Supplementary figures and images for: Role of the Default Mode Network in Cognitive Transitions
Source: Cereb Cortex. 2018 Jul 27;28(10):3685–96. doi: 10.1093/cercor/bhy167 (PMC6132281; doi:10.1093/cercor/bhy167)

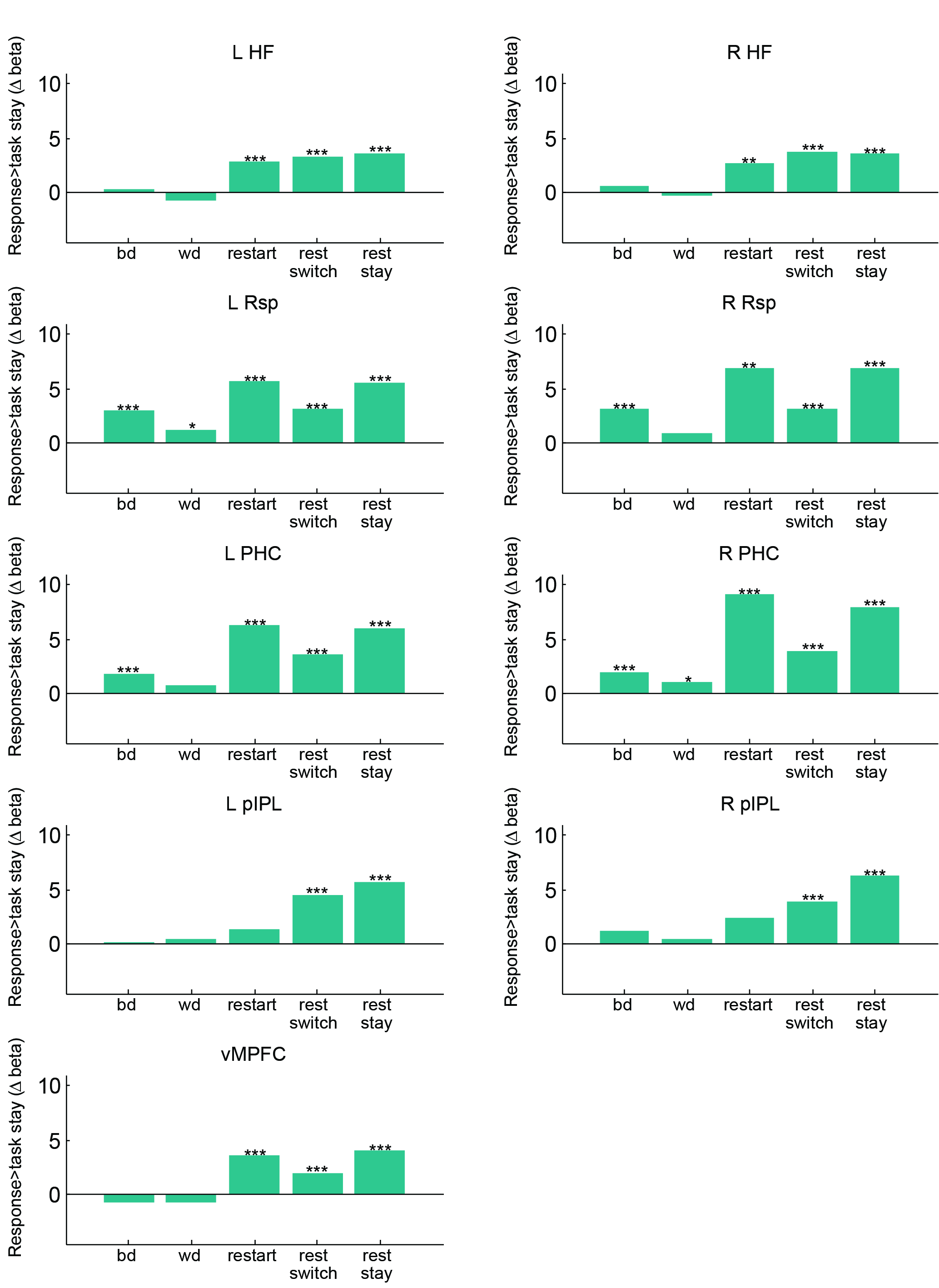

Supplement: Supplementary Data [file bhy167supp_1.zip › bhy167_SM_2.tif]

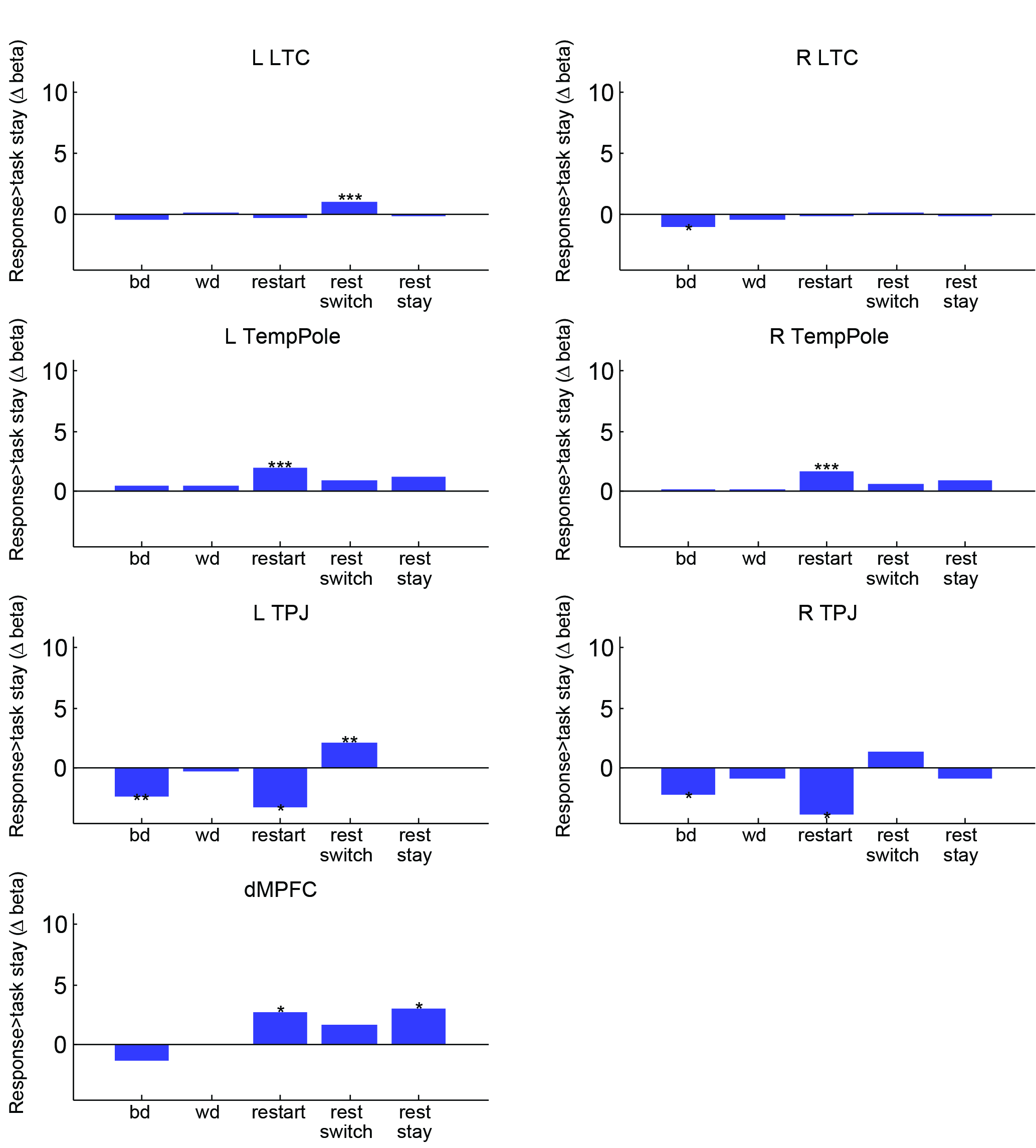

Supplement: Supplementary Data [file bhy167supp_1.zip › bhy167_SM_3.tif]

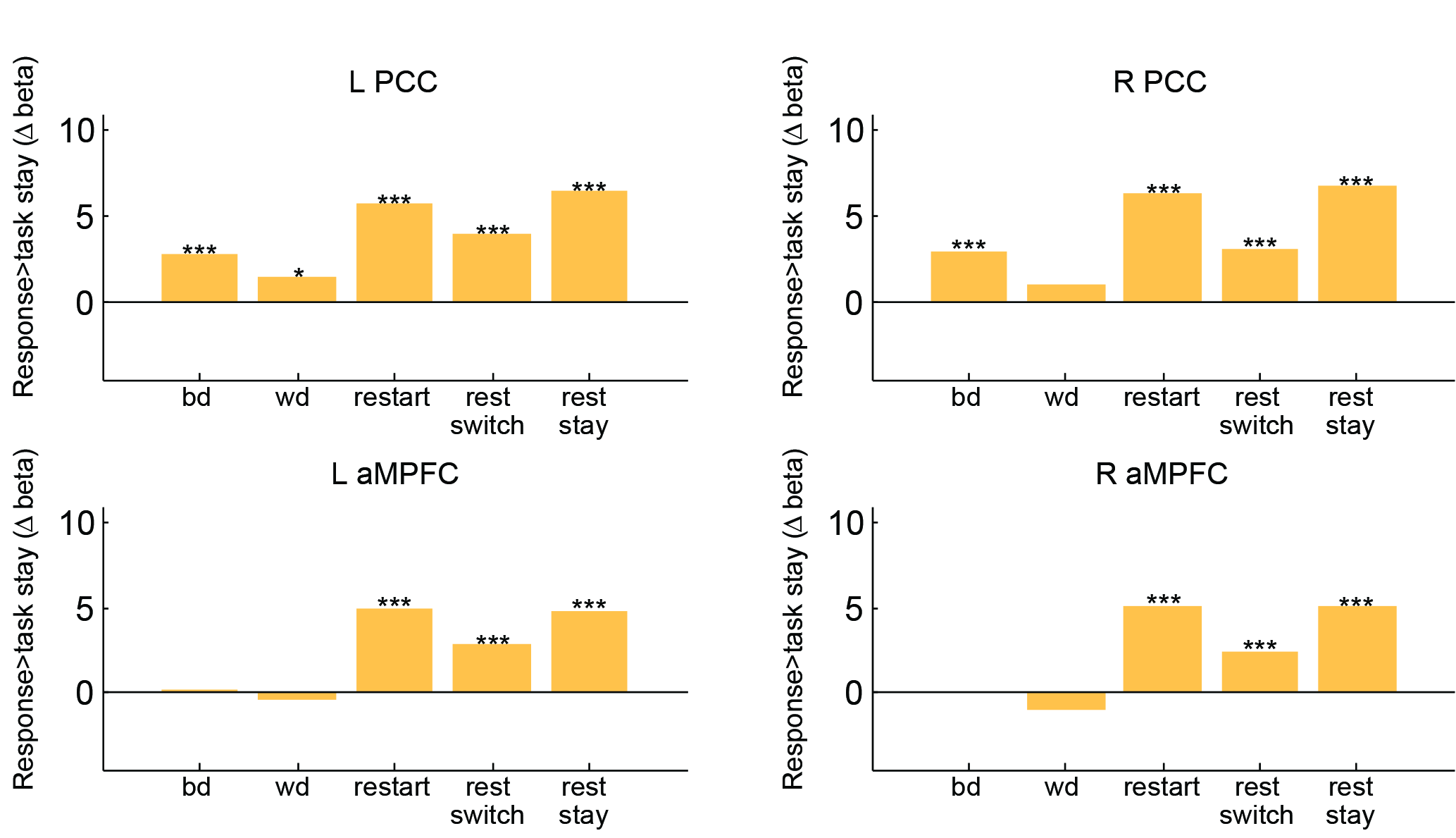

Supplement: Supplementary Data [file bhy167supp_1.zip › bhy167_SM_1.tif]
